# True Worth Recognized

**Published:** 1890-07

**Authors:** 


### TRUE WORTH RECOGNIZED.

The *Woman's Journal* says: "Miss Nellie Sanger, who is stenographer to President Harrison, and private secretary to his wife, was one of the young ladies asked to assist at the New Year's reception at the White House. On the same day, Miss Hunt, who is the daughter of a former cabinet officer and minister to Russia, but who, through reverses, has become wholly dependent upon herself, and is serving as secretary to Vice-President Morton's wife, was one of the most honored assistants at the reception in her employer's house. Both of these young ladies are clever, accomplished, and personally attractive, fitted to grace any drawing-room, but they are working women on salaries, and their formal appearance in official society is an innovation which is said to be without precedent. It is the pleasure of Mrs. Harrison and Mrs. Morton that the young ladies should rank socially with invited guests, and this decision is eminently sensible. It may be said to work an era in social progress when the 'better half' of an administration tacitly declares that a lady does not lose caste because she earns her living."

